# Contribution of ultrasonography to the diagnosis of internal bleeding in snakebite envenomation

**DOI:** 10.1186/s40409-016-0063-x

**Published:** 2016-03-16

**Authors:** Blaise Adelin Tchaou, Kofi-Mensa Savi de Tové, Yolande Sissinto-Savi de Tové, Aurélien Tchémaha C. Djomga, Abdou-Rahman Aguemon, Achille Massougbodji, Jean-Philippe Chippaux

**Affiliations:** Centre Hospitalier Universitaire et Départemental du Borgou/Alibori, Parakou, Bénin; Centre d’Étude et de Recherche sur le Paludisme Associé à la Mère et à l’Enfant, Cotonou, Bénin; Centre National Hospitalier et Universitaire Hubert Koutoukou MAGA, Cotonou, Bénin; UMR 216, Mère et enfant face aux infections tropicales, Institut de Recherche pour le Développement, Cotonou, Bénin; Faculté de Pharmacie, Université Paris Descartes, Sorbonne Paris Cité, Paris, France

**Keywords:** Envenomation, Ultrasound, Internal bleeding, Hemoperitoneum, Hematoma

## Abstract

**Background:**

In Africa, snakebite envenomations are frequently complicated by life-threatening hemorrhagic syndromes. The authors of the present study conducted a prospective analysis at the University Hospital of Parakou (north of Benin) for seven months (January 1 to July 31, 2014) to assess the contribution of ultrasonography to the diagnosis of internal bleedings and management of envenomation.

**Methods:**

An ultrasound examination was performed in all patients with clinical envenomation regardless of its severity. The study involved 32 patients admitted to the ICU of the University Hospital of Parakou.

**Results:**

The average age was 27 ± 13.9 years. The main signs of severity were: prolongation of clotting time (88 %), severe anemia (41 %), clinical hemorrhage (47 %), and shock (19 %). The ultrasound imaging showed internal hemorrhage in 18 patients (56 %). There were hematomas (22 %), hemoperitoneum (13 %) or a combination of both (22 %). The occurrence of internal bleeding and hemoperitoneum were mainly related to the delay of hospital presentation (*p* = 0.007) and the existence of external bleeding (*p* = 0.04). Thirty patients (94 %) received antivenom. Case fatality rate was 3.1 %.

**Conclusion:**

Ultrasonography may help in diagnosing internal bleeding, even in patients that did not show external hemorrhages, and evaluating its importance. As a consequence, the management of snakebite victims may be significantly improved.

## Background

Snakebite envenomations are an actual neglected public health issue in tropical regions, specifically in northern Benin, because of their high incidence and severity of clinical presentations [1, 2]. In northern Benin, over 70 % of envenomations are due to *Echis ocellatus* (West African carpet viper), a small and particularly abundant viper whose venom contains hemorrhagins and prothrombin activators that provoke severe bleedings [2]. The venoms from other species found in this region have limited hemorrhagic effect, even the common viper *Bitis arietans* (puff adder), whose bite results more often in severe necrosis rather than hematological symptoms.

The management of snakebite envenomation is made difficult not only by the low availability of antivenom in areas with high incidence of bites, but also by its high cost (25,000 CFA francs or 50 USD) [3, 4]. Without proper treatment, the victims resort to traditional medicine first, resulting in a considerable delay in hospital presentation that may cause serious complications, including hemorrhage [5]. Internal bleeding generally affect cavities and soft tissues of the organism (dermis, muscle, deep organs). Their clinical diagnosis is difficult and they often cause death, particularly in remote health centers where most envenomations occur [6]. It is therefore essential to diagnose them early, including with the use of medical imaging exams such as ultrasonography, a non-invasive, reproducible and relatively accessible test.

The objective of this study was to assess the interest of ultrasonography in the diagnosis and treatment of internal hemorrhage and its contribution to the management of snakebite envenomation particularly in health facilities deprived of hematology laboratory.

## Methods

The study was performed in the Intensive Care Unit (ICU) and Department of Medical Imaging of the University Hospital of Parakou (UHP) in North Benin. The prospective cross-sectional study was conducted over a period of seven months (January 1 to July 31, 2014). It received the approval of the institutional ethics committee.

The study population consisted of patients of both genders, snakebite victims, admitted to the host unit and transferred to ICU. Moreover, patients came from various health centers deprived of laboratory and medical imaging units. All patients with signs of envenomation regardless of the severity were included in the study. Patients underwent an ultrasound examination on admission. The choice of the site of the ultrasound exploration was oriented by clinical examination (anemia, shock, shifting dullness, painful swelling).

Ultrasonography was performed by a radiologist using a convex variable frequency probe (2.5–3.5 MHz) and a surface probe, also of variable frequency (5–10 MHz). Abdominal exploration was achieved through a convex probe to view the various peritoneal recesses. Any hemorrhagic fluid collections were sought after and quantified using the method of McKenney et al. [7]. Hemorrhagic nature of the collection was confirmed according to the presence of fine echoes in shifting dullness. Soft tissues were explored. Heart and lung exploration looking for possible pericardial and pleural effusions was performed. The presence of intestinal parietal hematoma was also sought.

The positive likelihood ratio (LR+), which is used for assessing the value of performing a diagnostic test, is a ratio based on the sensitivity and specificity of the test, in the present study, the ultrasonography results were compared with clinical findings [LR + = sensitivity/(1 – specificity)].

Data collection was based on register of hospital admission, case report form used in ICU and ultrasound findings. A standardized form was established for the analysis. The variables studied were: sociodemographics, clinical and biological parameters, therapy and evolution, and prognosis.

A few biological and hematological tests have been performed at patient presentation: blood count in 29 patients, whole blood clotting time in 30 patients, serum creatinine and urea levels in 11 patients.

Data analysis was performed using the software Epi Info® version 3.5.1. Statistical tests (*χ*^2^ and Fischer test) were used to detect any dependence between variables. The level of significance was *p* = 0.05.

## Results

In seven months, 308 patients were admitted to the ICU and among them, 32 cases of snakebite envenomation (10.4 %) were treated. The average age of patients was 27 ± 13.9 years and the median age was 27 years, ranging from 10 to 70 years. Most patients (59 %) were aged between 16 and 35 years, with a predominance of males (69 %) and a sex ratio of 2.2. In our series, 53 % of the victims were shepherds. Other identified activities were: housewives (19 %), students and pupils (19 %), and craftsmen (9 %). Snakebites occurred in the fields (75 %) when farming, during walking on roads (16 %) and at home (9 %).

The mean and median time to hospital presentation was 41 h and 40 h respectively, ranging from 6 to 96 h. Five patients (16 %) were referred from another health center and 27 (84 %) were admitted directly at emergency ward of UHP. Bite sites were foot (88 %), hand (9 %) and leg (3 %). Previous treatment administered to patients before UHP admission were: traditional treatment (44 %), antivenom therapy (9 %), association of traditional treatment and antivenom therapy (6 %). No treatment was applied in 13 patients (41 %). Traditional treatment consisted of scarification (29 %), applying black stone (21 %), administration of potions and herbal drinks (21 %), association of scarification and black stone (14 %). The composition of the treatment was not specified in 14 % of cases.

Edema was limited in 13 patients (41 %), locoregional in 18 (56 %) and generalized in 1 (3 %). Systemic hemorrhagic syndrome was present in 15 patients (47 %) who had: hematemesis (47 %), epistaxis (33 %) and gingival bleedings (13 %). The type of external hemorrhage was not reported in 7 % of the cases. Local bleeding from fang marks occurred in about 30 % of the victims, but they were not taken into account. The main signs of severity were blood coagulation disorders (88 %), systemic hemorrhagic syndrome (47 %), severe anemia (41 %), shock (19 %), hemoglobinuria (19 %) and skin necrosis (6 %).

**Fig. 1:**
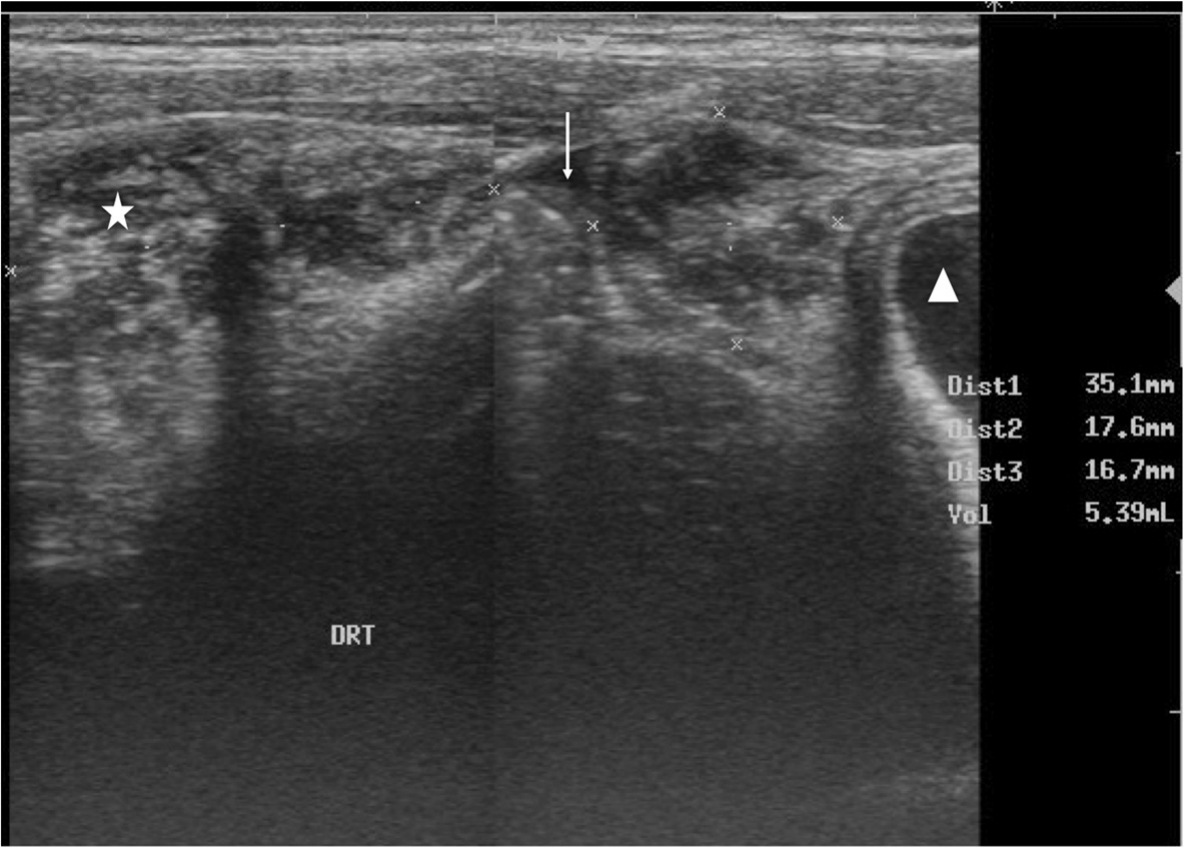
Ultrasound image of **a** longitudinal and **b** transverse section of the right iliac fossa. Adjacent to the bladder (triangle) there is a heterogeneous echogenic appearance of the psoas muscle (star) due to a hematoma associated with a discrete liquid collection (arrow)

**Fig. 2:**
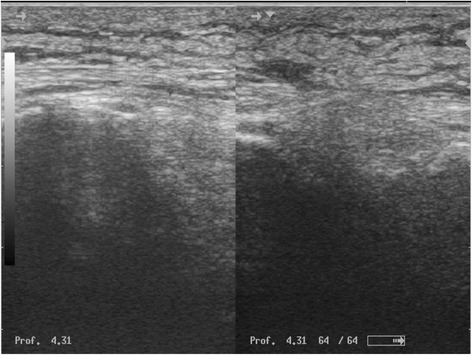
Ultrasound image of the soft parts of the leg. Thickening of the soft tissue with an echogenic laminated appearance

**Fig. 3:**
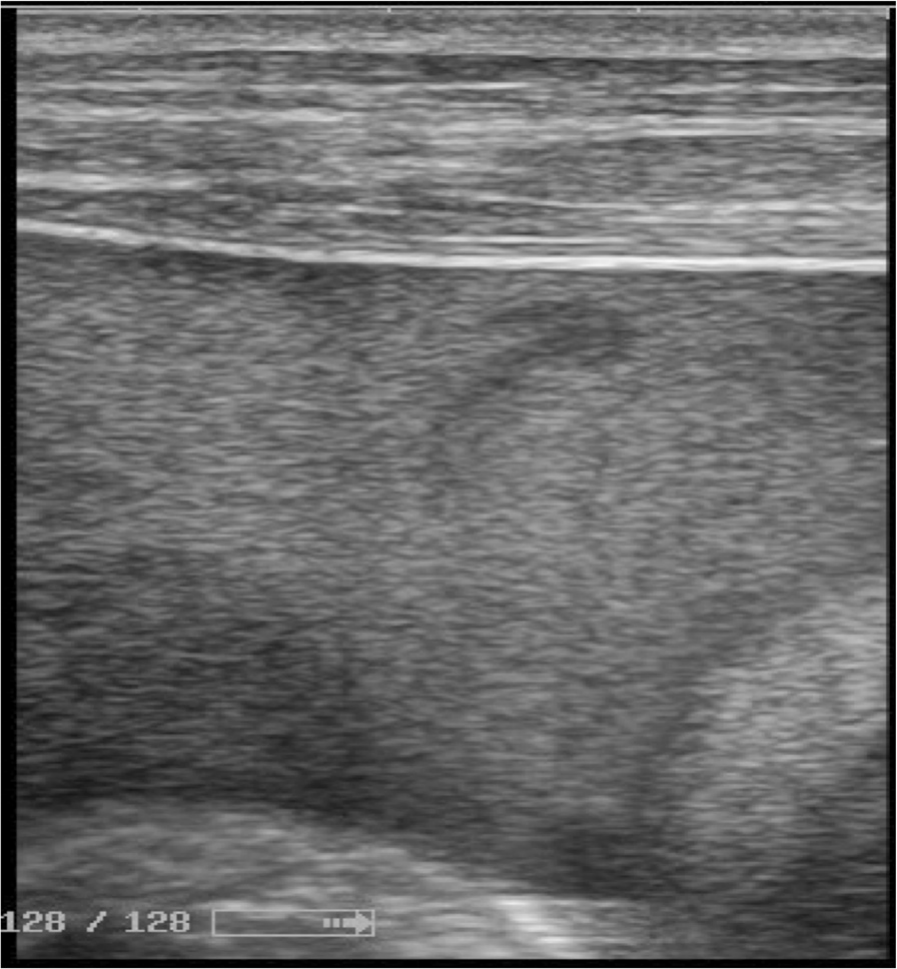
Ultrasound image of an intraperitoneal effusion containing multiple mobile echoes emphasizing its hematic origin

**Fig. 4:**
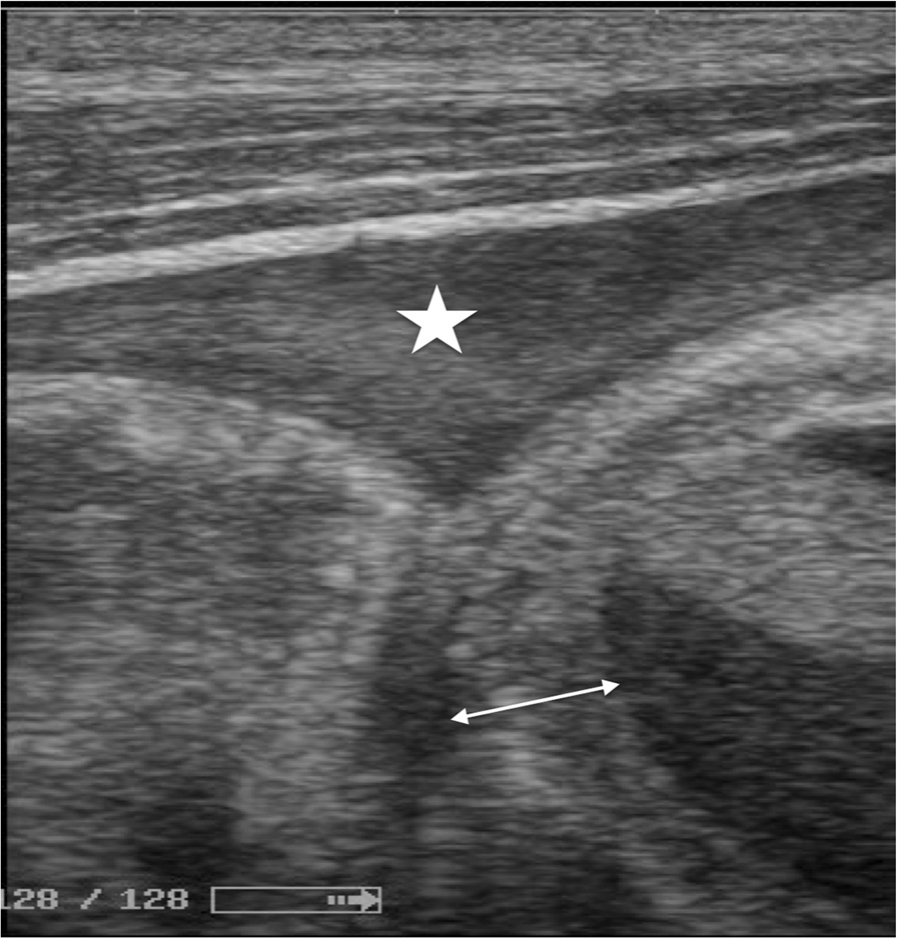
Ultrasound image of a hemoperitoneum (star) in the right paracolic gutter with thickened colonic walls suggesting a parietal hematoma (double-headed arrow)

**Fig. 5:**
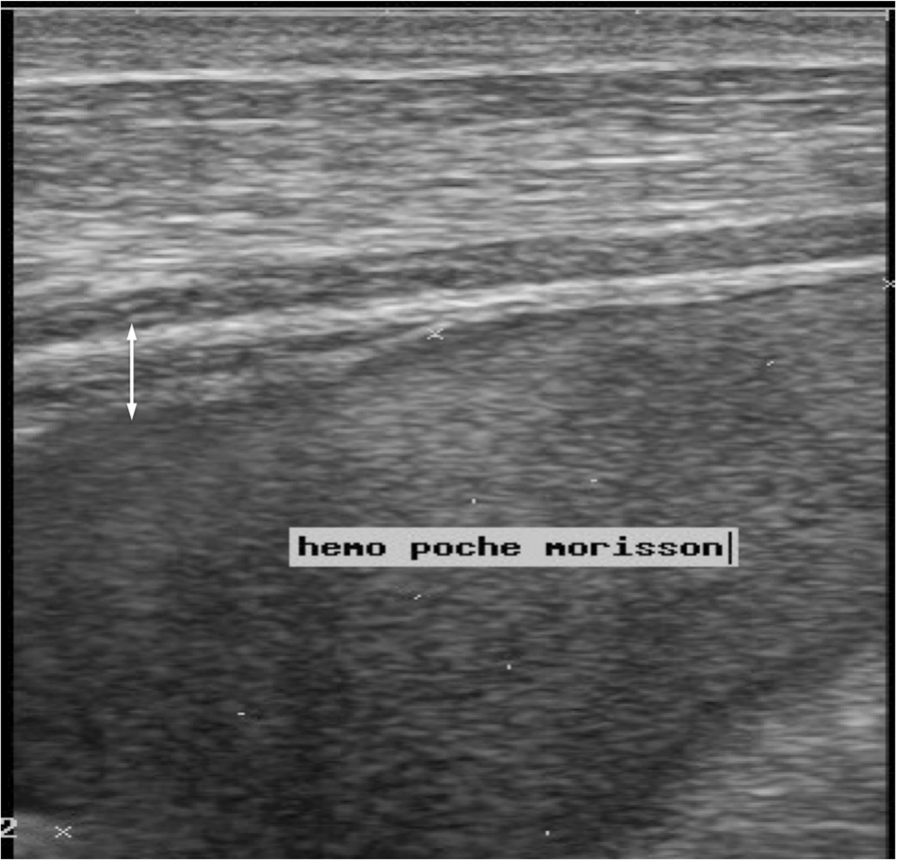
Ultrasound image of a hemoperitoneum in the pouch of Morrison with echogenic appearance of parietal peritoneum thickening (double-headed arrow)

Ultrasonography was performed in all patients and allowed to diagnose internal hemorrhage in 18 patients (56 %) showing either hematoma or hemoperitoneum, or both (Table 1 and 2). Abdominal hematoma was diagnosed in 14 patients (44 %) with various locations and volumes (Table 3). McKenney score was higher than 3 in three (27 %) patients out of 11 showing hemoperitoneum at ultrasonography. Examples of ultrasonography images are presented in Figs. 1, 2, 3, 4 and 5. Although external bleeding was significantly related to the hemorrhagic syndrome [sensitivity = 77.8 % (*CI* = 95 %; 56–99.6) and specificity = 92.9 % (*CI* = 95 %, 78.9–100); positive predictive value = 93.3 % and negative predictive value = 76.5 %], internal bleeding was diagnosed in four patients (13 %) that did not show external bleeding (Table 4). Finally, the LR+ obtained from the data presented in Table 4 was 10.94.

**Table 1:** Individual data regarding clinical bleeding, ultrasonography imaging, renal failure and treatment (*NA* = not available)

Patient number (sex, age)	Time before presentation	Clinical hemorrhage	USG signs	Internal blood volume (mL)	Antivenom doses	Renal failure
1 (M, 12)	6–24 h	Yes	Yes	2000	2	No
2 (M, 21)	6–24 h	No	Yes	190	1	No
3 (M, 19)	6–24 h	No	No	0	2	No
4 (M, 40)	>72 h	Yes	Yes	58	4	Yes
5 (F, 26)	6–24 h	Yes	Yes	90	2	No
6 (F, 13)	>72 h	Yes	Yes	NA	1	No
7 (F, 12)	<6 h	Yes	Yes	12	2	No
8 (F, 40)	24–48 h	No	Yes	NA	1	No
9 (M, 33)	>72 h	No	No	65	1	No
10 (F, 25)	48–72 h	No	Yes	NA	0	No
11 (M, 70)	<6 h	No	No	1200	1	No
12 (M, 10)	<6 h	Yes	Yes	NA	2	No
13 (M, 12)	6–24 h	Yes	No	72	1	No
14 (M, 30)	>72 h	Yes	Yes	NA	3	Yes
15 (M, 30)	>72 h	Yes	Yes	NA	3	No
16 (M, 35)	6–24 h	No	No	340	2	No
17 (F, 40)	48–72 h	Yes	Yes	13	3	No
18 (M, 32)	>72 h	No	No	0	1	No
19 (M, 40)	>72 h	Yes	No	0	2	No
20 (F, 35)	<6 h	Yes	No	0	1	No
21 (M, 28)	48–72 h	Yes	Yes	320	3	No
22 (M,17)	<6 h	No	No	0	1	No
23 (F, 30)	>72 h	No	Yes	NA	0	No
24 (F, 22)	6–24 h	No	No	0	1	No
25 (M, 24)	48–72 h	Yes	Yes	NA	2	No
26 (M, 10)	>72 h	No	Yes	184	2	No
27 (M, 19)	24–48 h	Hematuria	Yes	60	2	No
28 (F,28)	6–24 h	No	No	0	1	No
29 (M, 40)	24–48 h	No	No	0	1	No
30 (M, 28)	6–24 h	No	No	0	1	No
31 (M, 11)	>72 h	Yes	Yes	450	4	No
32 (M, 25)	>72 h	No	Yes	NA	2	No

**Table 2:** Distribution of patients according to the type of hemorrhage indicated by ultrasonography (percentages exceed 100 % because the same patient can have more than one symptom)

Type of internal bleeding	Number (%)
Hematoma	7 (22)
Hemoperitoneum	4 (12)
Hematoma + hemoperitoneum	7 (22)
Total	18 (56)

**Table 3:** Distribution of patients (*n* = 14) according to hematoma location and volume indicated by ultrasonography

		Number (%)
Location	Psoas sheath	5 (36)
	Paracolic gutter	1 (7)
	Rectoterin and psoas sheath	8 (57)
Volume	<100 mL	7 (50)
	100–500 mL	5 (36)
	>500 mL	2 (14)

**Table 4:** Distribution of patients showing internal hemorrhage diagnosed by ultrasonography according to the presence of bleeding at hospital presentation

		Hematoma and/or hemoperitoneum (%)		Total (%)
		Yes	No	
Bleedings	Yes	14 (44)	1 (3)	15 (47)
	No	4 (13)	13 (41)	17 (53)
Total		18 (56)	14 (44)	32 (100)

There is a high significant correlation (*p* = 0.007) between the presence of hemoperitoneum and time to hospital presentation (Table 5).

**Table 5:** Distribution of patients showing hemoperitoneum according to the time of hospital presentation

Time to hospital presentation	Hemoperitoneum (%)		Total (%)
	Yes	No	
<6 h	1 (3)	4 (13)	5 (16)
6–24 h	0 (0)	9 (28)	9 (28)
24–48 h	0 (0)	3 (9)	3 (9)
48–72 h	3 (9)	1 (3)	4 (13)
>72 h	7 (23)	4 (13)	11 (34)
Total	11 (34)	21 (66)	32 (100)

In our series, 30 patients (94 %) benefited from the antivenom treatment using the snake venom polyvalent antivenom (Vins Bioproducts Ltd, India). The average dose was 1.8 vials per patient (Table 6). The other elements of treatment were: pain relief (100 %), antibiotics (100 %), tetanus serum therapy (90 %), oxygen (79 %), transfusion (41 %), intravenous fluids (22 %) and surgery (9 %).

**Table 6:** Distribution of treated patients (*n* = 30) according to the number of antivenom doses

Number of doses	Number of patients (%)
1	13 (43)
2	11 (37)
3	4 (13)
4	2 (7)

**Fig. 6:**
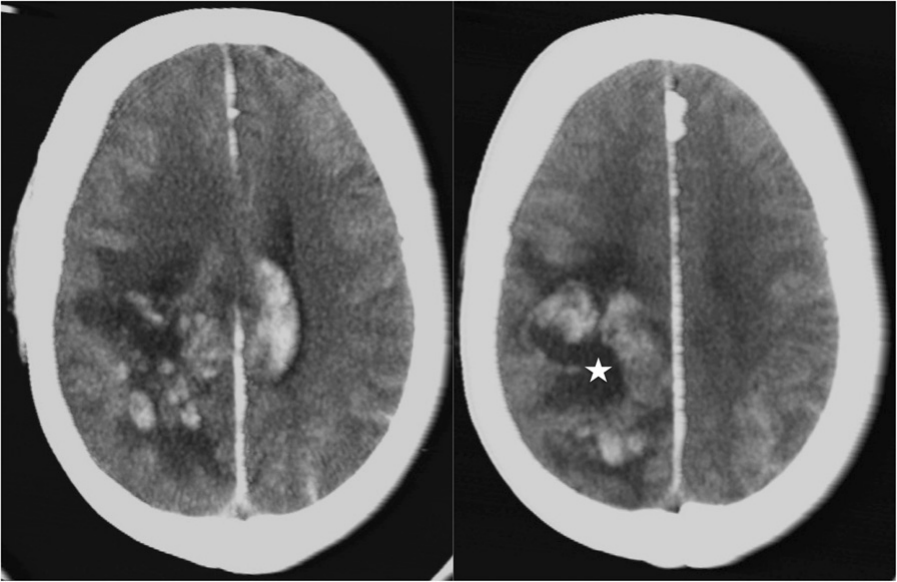
Axial CT scan without contrast medium injection. Spontaneous nodular hemorrhagic hyperdensity indicating establishment of an intraparenchymal hematoma with hypodense center (star). The right lateral ventricle is clear and the left is laminated. Note the presence of an associated subarachnoid hemorrhage

The average hospital stay in ICU was two days ranging from 1 to 4 days. One patient died (case fatality rate = 3 %) resulting from a coma due to a hemorrhagic stroke (Fig. 6). Twelve patients (38 %) were discharged from hospital against medical advice because they could not pay for care, but their condition was satisfactory. The various complications observed during hospitalization were: compartment syndrome in three patients (9 %), renal failure in two others (6 %) and hemorrhagic stroke (3 %) confirmed by CT scan without contrast.

## Discussion

The general profile of the patients corresponded to that of most studies conducted in the region [2–5, 8–12]. Most of them were young farmers who were bitten during field or herder works. The clinical presentation, in particular the distribution of bleeding symptoms, were similar to those described elsewhere [3, 4, 8–12].

The management also corresponded to what is generally observed in sub-Saharan Africa. The high frequency of antivenom treatment (94 %) should be highlighted, although the one used was probably not the most appropriate [12]. Hospital stay cannot be validly taken into account, since there is no standardized protocol in this regard and the decision was taken by the medical team based on their own experience and the patient’s condition. In addition, it should be noted that in sub-Saharan Africa discharge against medical advice is common, which poses the problem of evaluation of the management of snakebites.

Snakes were not identified in the present study. However, because of its abundance in the region and clinical findings, we can estimate that *Echis ocellatus* was responsible for most envenomations [5, 13]. The venom of this viper contains hemorrhagin, a metalloproteinase that induces deterioration of vascular endothelia, and ecarin, a prothrombin activator that provokes clot formation [14]. Subsequent bleedings result from two concomitant phenomena. On the one hand, hemorrhagins induce the extravasation of blood, and, then, ecarin provokes a systemic hemorrhagic syndrome, resulting from the consumption of plasma coagulation factors. If the first phenomenon is immediate and dose dependent, the second one is later (in the case of *E. ocellatus*, it can occur on the 2^nd^ day) and time dependent [14]. Nevertheless, both produce a diffuse hemorrhagic syndrome that only the appropriate antivenom administration can interrupt [11, 12, 15].

In the study, the average delay for hospital presentation is particularly high, more than in many other studies, but quite close to that reported by Drabo et al. [9] in Burkina Faso, which was 50 h [5]. This delay in the consultation is due to several factors: the difficulty of access to health care by patients living in rural areas, underestimate of the severity of the envenomation, and a preference for the traditional treatment both more accessible and supposed to be more responsive to the supernatural nature of the attack by a snake [16]. The use of modern medicine is considered after failure of traditional treatment. In addition, the UHP is the ultimate reference, making it the 3^rd^ or 4^th^ option as a therapeutic resource.

The delay in hospital presentation results in the onset of various complications: secondary infections, hemorrhage, necrosis, renal failure etc. It should be emphasized the possible influence of the delay of treatment in the constitution of internal blood collections that ultrasound is able to diagnose. The more treatment is delayed, the higher is the risk of an internal bleeding, which is suggested by the significant link between the incidence of internal hemorrhage and the delay of presentation (*p* = 0.007).

The lack of consensus on clinical gradation when this study was conducted did not allow us to assess the contribution of ultrasonography according to clinical gradation of edema. This could help in the indication of ultrasonography.

Obviously, considering the positive likelihood ratio of 10.94, external bleeding should prompt to perform ultrasonography. In other words, most patients with external bleeding (93 %) presented internal hemorrhage. Clinical hemorrhage, including hemoperitoneum, due to severe envenomation by viper bites was already described by Mensah et al. [6]. However, the sensitivity of ultrasonography should be regarded as quite poor (77.8 %). The surface probe permitted to complete the examination by studying the abdominal wall to show hematoma. It also allowed a better study of the right and left paracolic gutter. McKenney et al. [7] established a score that assesses the volume of a blood collection according to its localization in peritoneal recesses. The application of this score allowed to conclude that 27 % of patients showed a blood collection with more than two liters (score ≥ 3), a situation that can induce the occurrence of shock.

Nevertheless, ultrasonography performed in all envenomed patients, regardless of the severity of symptoms, allowed to reveal internal bleeding (hematoma and/or hemoperitoneum) in 56 % of patients, that is, up to 10 % of patients without external bleedings. This contributed to the diagnosis of previously unrecognized complications that could cause death.

## Conclusion

Ultrasonography showed a good capacity to diagnose internal bleeding of different degrees, even of small volume, both in patients with evident clinical bleeding disorders and those with deficient blood coagulation, but without any apparent clinical manifestation. This examination should be systematic – whenever possible – in all patients envenomed by vipers (i.e. showing at least an inflammatory syndrome) and whose blood clotting appears disturbed (particularly if whole blood clotting test is abnormal). A new study will allow us to include these criteria in the clinical gradation of envenomation.

### Ethics approval

The prospective cross-sectional study was approved by the Ethics Committee of the University Hospital of Parakou (UHP), Benin.
